# Obesity, Mediterranean Diet, and Public Health: A Vision of Obesity in the Mediterranean Context from a Sociocultural Perspective

**DOI:** 10.3390/ijerph18073715

**Published:** 2021-04-02

**Authors:** Francesc Xavier Medina, Josep M. Solé-Sedeno, Anna Bach-Faig, Alicia Aguilar-Martínez

**Affiliations:** 1 FoodLab & UNESCO Chair on Food, Culture and Development, Faculty of Health Sciences, Universitat Oberta de Catalunya (UOC), Rambla del Poblenou, 156, 08018 Barcelona, Spain; abachf@uoc.edu (A.B.-F.); aaguilarmart@uoc.edu (A.A.-M.); 2 Department of Obstetrics and Gynecology, Hospital del Mar, Universitat Autònoma de Barcelona (UAB), Passeig Marítim, 25-29, 08003 Barcelona, Spain; jsole@psmar.cat

**Keywords:** obesity, Mediterranean diet, public health, sociocultural perspective, social change

## Abstract

Obesity is a disease that straddles medico-nutritional, psychological, and socio-cultural boundaries. There is a clear relationship between lifestyle and obesity, and today the Mediterranean diet in the Mediterranean area may represent an interesting corrective asset. However, we should not be under any misapprehension about the model’s capacity for action in non-nutritional terms. Our societies are experiencing a process of rapid change, and the Mediterranean area is no exception. The aim of this article is to present a view of obesity in the Mediterranean context from an open, mainly socio-cultural perspective, but from different points of view (medical, nutritional), seeking points of convergence and elements that contribute to the understanding of and approach to the disease in the context of the Mediterranean diet. As a public health and a multidimensional social problem, obesity must be dealt with in a holistic, open, and cross-disciplinary manner to ensure that it can be understood coherently. The only way to keep the usefulness of the Mediterranean diet within desirable limits will be our societies’ vitality and interest in rapidly adapting the Mediterranean diet to social change, thus providing valid answers to today’s needs.

## 1. Introduction

Today, obesity is considered a highly prevalent and, in many cases, chronic disease. World Health Organization (WHO) projections estimated that worldwide, in 2016, approximately 1.9 billion adults were overweight and 600 million adults were obese. WHO specifically states that rates of overweight and obesity continue to grow worldwide. From 1975 to 2016, the prevalence of overweight or obese children and adolescents aged 5–19 years increased more than four-fold, from 4% to 18% globally [1,2].

From a clinical point of view, overweight and obesity occur when excess fat accumulation (regionally, globally, or both) increases risk to health. It is the point at which health risk is increased that is most important because, as covered below, body weights and fat distributions that lead to the expression of comorbid diseases occur at different thresholds depending on the population. On the other hand, obesity is a disease characterized by excess adiposity that is a source of extensive morbidity and mortality due to various weight-related complications. Therefore, the diagnostic evaluation should consist of an anthropometric measure that reflects increased fat mass and an indication of the degree to which the excess adiposity is adversely affecting the health of individual patients [3]. Overweight and obesity are both defined as abnormal or excessive fat accumulation that presents a risk to health [4].

We also have to point out that, from the bio-medical point of view, metabolic syndrome (MetS) is defined as a pathological condition characterized by abdominal obesity, insulin resistance, hypertension, and hyperlipidemia. The incidence of MetS has been closely associated with obesity, as visceral fat plays a critical role in MetS pathogenesis. The syndrome feeds into the spread of diseases such as type 2 diabetes, coronary diseases, stroke, and other disabilities. The total cost of the malady, including the cost of health care and loss of potential economic activity, is in the trillions [5].

The metabolic syndrome is a complex pathophysiological state that originates primarily from an imbalance of calorie intake and energy expenditure but is also affected by the genetic/epigenetic make up of an individual, the predominance of sedentary lifestyle over physical activity, and other factors such as the quality and composition of food and the composition of gut microbes. No single remedy can be prescribed for its eradication or even curtailment [6].

Obesity, at the same time, has substantial social and cultural, lifestyle, and dietary habit components. The condition of being or not being obese is one that affects individuals and societies across the board in terms of the construction of socio-cultural discourses (often laden with considerable moral significance) and, particularly in recent years, in terms of the analytical and action parameters developed in the field of public health.

From the beginning, it seems necessary to make it clear that, from a social point of view, but also from any broader point of view, obesity is not only due to high levels of caloric intake and low physical activity, but to multifactorial aspects that make it a highly complex fact: psychological factors, cultural aspects, sleeping habits, personal stress, kind of work and working hours, sedentary lifestyle, age, genes, diet, metabolism, hormones, dysbiosis, pathologies, drugs consumption, or individual complexion must be taken into account. In this regard, obesity is more than a simple choice of performing physical activity and eating better.

Regarding the general framework of the prevention of obesity, studies with diets that can reduce obesity rates must be taken into account. In this way, the scientific literature has extensively pointed out the beneficial effect of the Mediterranean diet on the health of the individual in terms of obesity. However, social factors have routinely been neglected in relation to studies of a more medical and nutritional nature.

Drawing on examples from today’s European Mediterranean context, the aim of this article is to review some of these issues from different points of view: medical and nutritional, psychological, and mainly socio-cultural, seeking points of convergence and elements of understanding and joint action.

## 2. Obesity as a Problem: Between the Individual and the Public, between the Biological and the Social

As a medical problem, obesity has been a focus of research for many decades. In the late 1970s, Garrow and Warwick [7] stated that obesity “is common enough to constitute one of the most important medical and public health problems of our time, whether we judge its importance by shorter expectation of life, increased morbidity, or costs to the community in terms of both money and anxiety”.

Today, obesity is considered one of the main causes of morbidity and mortality in Western countries, and its prevalence is increasing in both developed and developing countries. However, some authors have already drawn attention to the need to consider that the established relationship between obesity and disease varies from one population to the next, and that it should take account of various social and environmental aspects linked to different levels of modernization [8,9].

Following Mennell [10] and his “civilizing of appetite” concept as the social domestication of the way of eating, Gracia [11] has noted that, in the past 50 years, this process of civilizing has become more intense, thus giving rise to four different yet closely related phenomena: the establishment of an ideal body weight and dietary norms; the construction of thinness as an attribute of health and of social distinction; the recognition of obesity as a disease; and the transformation of health and the body into socio-economic factors and, therefore, into business opportunities.

Regarding the above-mentioned phenomena, we could add that the first two have also had a significant influence over the creation of an ideal “Western” aesthetic concept, which extols thinness as the model to be followed and establishes the cultural norm of what, in our societies, an attractive (or “normative”) body is or is not [12,13,14]. In turn, such a process fosters a conflict of a psycho-social nature in individuals who do not meet that norm. De Garine [15] stated that the established body concept is very hard to achieve and maintain in the framework of lifestyles like those we now find in our urban and industrialized contexts; and such situations lead individuals to a pathological and culturally stigmatized state that is difficult to overcome. As Counihan [16] has also indicated, “in western society, obesity is unequivocally negative: in terms of aesthetics, morality, and health”.

Several elements converge in this reflection. Today, we are faced with a process that makes us individually responsible for disease and its social costs [17]. In this respect, we can see how an individual who deviates from the norm, when he/she has the potential to choose, is made socially responsible for his/her disease. Why? Because the individual has not done everything within his/her power to prevent it, so as not to become an economic cost to the public health system [17].

Spanish sociologist Enrique Luque [18] asks certain questions: Who is responsible for the world pandemic of obesity declared by the WHO? Is it an individual problem whose solution is based on education, or is it also (mainly?) a public issue, of a structural nature? This author argues that the general trend toward obesity reveals some of the contradictions of a pathological agri-food system that has shaped an “obesogenic” food environment. The interests of the industry in the wide distribution of some products, subsidies for overproduction, and the fragile role of the consumer in the context of powerful marketing pressures should be some of the issues to have in mind.

On the one hand, an individual’s potential to choose his or her type of food therefore sets the rule of social behavior (mainly urban) and the development of public health policies on nutrition. However, some experts have underscored the fact that socio-economic, education, and information access differences can clearly limit the ability to choose appropriately [19,20,21,22,23].

On the other hand, today it is almost needless to recall that the economic, labor, social, and demographic changes of recent decades have led to changes in diet and lifestyles, which have affected the very foundations of the way we live and eat—and obesogenic societies are also the result of that.

Today, lower levels of physical activity [24] and higher levels of calorie intake [25] are two of the main causes of obesity, since they lead to an energy surplus. Other factors do have an impact, however, such as genetic makeup and gut flora, which explain differences between individuals in terms of energy expenditure and the ability to store energy in response to nutrients in the diet [26,27,28].

From a medical viewpoint, morbidity goes beyond chronic diseases such as diabetes, high blood pressure, and cardiovascular disorders [28,29]. It is also an important risk factor for many types of cancer [30,31,32,33], and it has an impact on how the treatment of cancer is planned, owing to the surgical risks involved [34,35,36]. Even the prognosis of infectious diseases, such as the recent COVID-19, can be worse because of obesity [37].

Obesity also leads to a significant deterioration in functional capacity and health-related quality of life, and there seems to be a direct proportional relationship between body mass index (BMI) and the degree of quality-of-life deterioration [38,39,40,41]. While some authors have suggested that obesity is perceived, by those affected by it, as a problem that has a greater impact on physical functioning (activity limitation, skeletal muscle problems, respiratory problems, body pain, etc.) than on psycho-social aspects (depression, body dissatisfaction, low self-esteem, quality of sexual life, etc.), specific action on the latter has a direct impact on both general quality of life and medical problems [42]. Actions of this type are particularly necessary in obese individuals because they are the ones who acknowledge that they are the most psycho-socially affected [38,40]. Thus, obese people are today faced with the challenge of losing weight to improve not only their physical problems but also many aspects of their psycho-social problems [42].

## 3. The Mediterranean Area: A Particular Context?

The history of the Mediterranean diet concept is relatively recent yet very intense, but we shall not go into that here. All we want to underscore is that it has led to the parallel development of two explanatory discourses: one that is sociocultural and another that is medical-nutritional. Each of the two discourses respond to very different interests and, until fairly recently, it was nigh on impossible for them to engage in an open, productive dialogue. UNESCO’s designation of the Mediterranean diet as Intangible Cultural Heritage of Humanity in 2010 (and the preparation of the nomination in previous years) was a unique opportunity for there to be a dialogue between the two perspectives [43,44,45].

In that designation, it is acknowledged as a way of life (using the original meaning of the Greek word *daiata*, which means exactly that: way of life) [46]. In this regard, the relationship between lifestyles and obesity—understood as a public health problem—is clear. Today, the Mediterranean diet in the Mediterranean area may represent an interesting corrective asset [47]. As different authors have pointed out, obese individuals undergoing medical nutritional treatment based on the Mediterranean diet substantially decrease their body weight and other anthropometric measurements used to define the presence of obesity [48,49].

On various occasions, the Mediterranean diet has been held up as a healthy lifestyle in relation to different aspects of public health, among which is obesity, underscoring its balance between energy density and nutrient richness (food in relation to energy expenditure); its content in low-glycemic-index and high-fiber foods; and particularly frugalness, moderation, and maintaining an active lifestyle [50,51].

However, we should not be under any misapprehension about the model’s capacity for action in non-nutritional terms. While the Mediterranean diet is considered today as a common heritage inscribed by UNESCO [45,52] that can be found in different degrees in the Mediterranean societies, it is also true, as mentioned earlier, that our societies are experiencing a process of rapid, destructuring change—and the Mediterranean is no exception. In this regard, for example, adherence to this dietary pattern is not found any more in a high percentage of individuals. On the other hand, the cuisines that are nowadays considered traditional to those countries are not totally equal to the old traditional cuisines, considered even today as characteristic or paradigmatic of the Mediterranean diet. Change and evolution are inherent aspects of culture, and while we may be able to have some influence over the direction change takes, the complexity of forces and interests is also considerable [53].

Low adherence to the Mediterranean diet has been related to the so-called current obesity epidemic in the south of Europe. Current dietary habits in the Mediterranean countries are moving towards a Western dietary pattern that is rich in saturated fats and refined carbohydrates, low in quality, and high in calorie intake [54]. It is considered a paradox that the countries with a higher life expectancy in the context of a reference dietary prudent health pattern actually have such obesity figures [55].

Specifically, in Spain, but generally speaking in several Mediterranean countries, the nutrition transition in recent years has resulted in a reduction in the consumption of cereals, potatoes, and legumes, together with a rise in meat and processed meat and non-alcoholic drinks [55]. From a nutritional point of view, these dietary changes translate into an increase in saturated fats and proteins of animal origin (especially those of animal origin), while complex carbohydrates decrease (along with a higher consumption of total and added sugars). The sedentary lifestyles of the Mediterranean countries have been raised as one of the main drivers related to the prevalence of obesity, both in childhood and adulthood, together with the dietary changes mentioned.

Adopting those habits has been linked to obesity in the obesogenic environments, mostly in low-income and in more urban areas [56]. Palatable energy-dense food can lead to overconsumption through the disruption of appetite regulation [57]. Meanwhile, plant-rich diets such as the Mediterranean diet are high in dietary fiber, which, through several mechanisms, contribute to satiation and satiety, which are key in appetite regulation [58]. To reduce Western diet exposure and increase adherence to the Mediterranean diet, knowledge and culinary skills should be provided to the younger generations [59,60].

In countries with a Mediterranean tradition such as Spain, obesity has reached a prevalence rate of around 25%, one of the highest in Europe [61,62,63]. Specific studies on child obesity confirm this growing trend and reveal a greater prevalence in the southern zone of Europe (up to 20%) than in the northern zone (less than 5%). At the top of the list are Mediterranean countries including Italy and Cyprus [64]. This rise in the prevalence of obesity coincides with the diminishing adherence to the Mediterranean diet model since the 1960s [65,66], which in turn coincides with Mediterranean societies’ access to modernity, with marked improvements in their respective socio-economic situations. In this respect, it should first be noted that, in the historically (even recent) cultural contexts of the majority of such societies (at least until the middle of the 20th century, when the problems of hunger and malnutrition were the most frequent and obesity was little more than anecdotal and with a strong component of social class), malnutrition problems were widespread and fatness was even considered aesthetically positive [67,68]. Secondly, if an element is incapable of finding its place in a particular culinary system, then it will never really become part of that system. Thus, the potential of the Mediterranean diet—as a lifestyle and, therefore, as a model that still encompasses a set of know-how, preparations, and habits that continue to be valid—is very important [69]. It must also be added that a good part of the cultural aspects that may have some kind of influence have been either little studied (or not studied at all), or well neglected. For example, some authors have pointed out the possible positive influence of aspects such as commensality in relation to the consumption of a more adequate and healthy diet, or even with a certain decrease in obesity rates [70,71,72,73,74,75].

However, the only way to keep the usefulness of the Mediterranean diet within desirable limits will be our societies’ vitality and interest in rapidly adapting the Mediterranean diet to social change, thus providing valid answers to today’s needs. Change is inevitable, though influence can be exerted over the direction it takes.

Consequently, as a public health and a multidimensional social problem, obesity—in general, and in the Mediterranean area in particular—must be dealt with in a holistic, open, and cross-disciplinary manner to ensure that it can be understood coherently.

## 4. Conclusions: The Need for Cross-Disciplinary Views

Human nutrition is far from a one-dimensional affair. As the British anthropologist Mary Douglas [76] stated more than forty years ago, “No human activity more puzzlingly crosses the division between nature and culture than the selection of food. It is part of the nurture of the body, but it is also very much a social matter”.

Its complexity requires different views, different perspectives, and different levels of comprehension. So, as some authors from social perspectives [77,78] have suggested, a cross-disciplinary approach is required—one that enables an integrative and comprehensive perspective.

As a bio-medical and socio-cultural object of study, obesity has neither a single cause nor a single approach to its analysis. Furthermore, as an object of study it varies enormously depending on the level of analysis performed: global or local, general or intra-social, micro or macro-economic, micro or macro-structural, etc. Despite growing globalization, obesity does not affect every population in the same way. Likewise, not all fat people are ill and not all of us eat badly [79].

From a public health viewpoint, such dimensions matter. The articulation of preventive campaigns and/or therapeutic actions, their impact, and their consequences depend on these premises. Attacking a problem as a whole by targeting just one of its components would be nonsensical. We ask ourselves to what extent this model—however rational— is proving efficient. It cannot fail to come as a surprise that the rate of obesity has increased despite the fact that health authorities have spent many decades trying to educate people about healthy lifestyle habits, and the fact that the population has a good knowledge of nutrition recommendations. What, then, is the point of so many (costly) actions [80]?

Even in the Mediterranean area, where many studies are still talking about an idealized diet anchored in the years before World War II [81], the only way to keep the usefulness of the Mediterranean diet within desirable limits is by providing valid answers to today’s needs from different perspectives and disciplines, by including all social actors that come into play, and by not only discussing health recommendations that are difficult to enforce due to present social change and constraints.

It may be the case that no further studies on diets are needed to deal with obesity, but rather a clear and open commitment to its prevention. In this respect, it is crucial for nutrition education to include some paradigm changes and for the social, economic, and cultural reality of its protagonists to be taken into account to improve adherence to the practices proposed [42]. As a public health problem, obesity therefore needs complementary points of view offering the most complete and comprehensive vision possible of a clearly complex phenomenon.

## Data Availability

Not applicable.
